# Differential cellular origins of the extracellular matrix of tumor and normal tissues according to colorectal cancer subtypes

**DOI:** 10.1038/s41416-025-02964-z

**Published:** 2025-03-03

**Authors:** Hyun Jin Lee, Sang Woo Park, Jun Hyeong Lee, Shin Young Chang, Sang Mi Oh, Siwon Mun, Junho Kang, Jong-Eun Park, Jung Kyoon Choi, Tae Il Kim, Jin Young Kim, Pilnam Kim

**Affiliations:** 1 Department of Bio and Brain Engineering, KAIST, Daejeon, 34141 Republic of Korea; 2 Korea Research Center for Bioconvergence Analysis, Korea Basic Science Institute, Ochang, 28119 Republic of Korea; 3 Department of Internal Medicine, Institute of Gastroenterology, Brain Korea 21 Project for Medical Science, Severance Hospital, Yonsei University College of Medicine, Seoul, 03722 Republic of Korea; 4 Graduate School of Medical Science and Engineering, KAIST, Daejeon, Republic of Korea; 5 SCL-KAIST Institute of Translational Research, KAIST, Daejeon, Republic of Korea

**Keywords:** Cancer microenvironment, Colorectal cancer, Tumour heterogeneity, Proteome informatics

## Abstract

**Background:**

Understanding the proteomic-level heterogeneity of the tumor microenvironment (TME) in colorectal cancer (CRC) is crucial due to its well-known heterogeneity. While heterogenous CRC has been extensively characterized at the molecular subtype level, research into the functional heterogeneity of fibroblasts, particularly their relationship with extracellular matrix (ECM) alterations, remains limited. Addressing this gap is essential for a comprehensive understanding of CRC progression and the development of targeted therapies.

**Methods:**

24 tissue samples from 21 CRC patients, along with adjacent normal tissues (NAT), were collected and decellularized using a detergent-based method to enrich the ECM component. Proteomic analysis of ECM-enriched samples was performed using tandem mass tag (TMT) spectrometry, followed by statistical analysis including differential expression protein (DEP) analysis. Single-cell RNA sequencing (scRNA-Seq) data from public datasets were integrated and analyzed to delineate cell states within the TME. Bulk tissue RNA-Seq and bioinformatics analysis, including consensus molecular subtype (CMS) classification and single-cell level deconvolution of TCGA bulk RNA-seq data, were conducted to further explore gene expression patterns and TME composition.

**Results:**

Differential cellular origin of the NAT and tumorous ECM proteins were identified, revealing 110 ECM proteins enriched in NAT and 28 ECM proteins in tumor tissues. Desmoplastic and *WNT5A*^+^ inflammatory fibroblasts were indicated as the sources of tumor-enriched ECM proteins, while *ADAMDEC1*^+^ expressing fibroblasts and *PI16*^+^ expressing fibroblast were identified as the sources of NAT-enriched ECM proteins. Deconvolution of bulk RNA-seq of CRC tissues discriminated CMS-specific fibroblast state, reflecting the biological traits of each CMS subtype. Specially, seven ECM genes specific to mesenchymal subtype (CMS4), including *PI16*^+^ fibroblast-related 4 genes (*SFRP2, PRELP, OGN, SRPX*) and desmoplastic fibroblast-related 3 genes (*THBS2, CTHRC1, BGN*), showed a significant association with poorer survival in patient with CRC.

**Conclusion:**

We conducted an extracellular matrix (ECM)-focused profiling of the TME by integrating quantitative proteomics with single-cell RNA sequencing (scRNA-seq) data from CRC patients. We identified the ECM proteins of NAT and tumor tissue, and established a cell-matrisome database. We defined mesenchymal subtype-specific molecules associated with specific fibroblast subtypes showing a significant association with poorer survival in patients with CRC. Our ECM-focused profiling of tumor stroma provides new insights as indicators for biological processes and clinical endpoints.

## Introduction

Colorectal cancer (CRC) progresses through a series of stages, marked by the accumulation of mutations in colonic epithelial cells [1]. The cancer cells generally become more heterogeneous, which in turn promotes to diversifies the local tumor microenvironment (TME). The local TME cues have a crucial role in possessing unique molecular signatures and varying levels of treatment sensitivity; therefore, an accurate assessment of TME heterogeneity is essential for enabling precision medicine [2].

Single-cell RNA sequencing (scRNA-seq) analysis has greatly enhanced our understanding of the TME and its cellular diversity [3]. In CRC, scRNA-seq analysis has successfully distinguished the cellular composition of the TME [4]. Moreover, the identification of functionally diverse and heterogeneous stromal cell types has led to a broader recognition of TME heterogeneity [5]. Recent research among stromal cells has underscored the significant importance of the phenotypic plasticity of fibroblasts in the ecosystem of the TME. The fibroblasts are the primary source of acellular components, such as the extracellular matrix (ECM), which plays a crucial role in the alterations of tissue integrity and homeostasis during cancer progression [6, 7]. Although the inter-tumoral heterogeneity within CRC have been characterized based on the diverse functional roles of fibroblasts [8], there is limited research focusing on the functional heterogeneity of fibroblasts linked to alterations in the ECM.

Consensus molecular subtypes (CMS) have been developed to classify CRCs based on tissue transcriptomics and study their corresponding TMEs [9]. CMS4, known as mesenchymal subtype, has distinct phenotypic features compared to the other CMS types. Recent research has reported that the CMS4 is characterized by extensive matrix remodeling, showing a high fibroblast content [10]. So far, there has been no comprehensive analysis or research on the characteristics of fibroblasts according to the CMS, especially regarding the distinct stromal features of CRC subtypes like CMS4. Furthermore, studies on the characteristics of the ECM differentiating between the CMS4 and other subtypes are completely lacking.

In this study, to develop a comprehensive understanding of the stroma heterogeneity in the molecularly heterogenous colorectal cancer tissues, we conducted an ECM-focused proteomic analysis alongside a stromal cell-focused single-cell analysis. We profiled the ECM-focused stromal features in ECM-enriched samples isolated from human surgical specimens of CRC using a tandem mass tag (TMT)-based approach. By integrating ECM proteome data with single-cell RNA sequencing and deconvolution of bulk-RNA sequencing data, we identified the differential cellular origins of ECM proteins across normal adjacent to tumor (NAT)/CRC/specific CRC subtypes. As previous researches have not clearly defined subtype-associated ECM and stromal cell subtype, our research specifically targeted the mesenchymal subtype, CMS4, and identified distinct stromal characteristics unique to this subtype.

## Materials and methods

### Patient and tissue sample collection

This study included 21 patients with CRC who were diagnosed on the basis of colonoscopy findings, underwent surgery from Jun. 2019 to May. 2020, and provided bulk RNA sequencing data from their tumor tissues (Supplementary Table 1A). Paired CRC samples and NAT samples were obtained from three patients. After surgical resection, tissue samples from the tumor and NAT tissues were collected and immediately transferred for tissue preparation. Additionally, 13 CRC and 5 NAT samples were obtained from 13 and 5 patients, respectively. Patient demographics and clinical characteristics were recorded based on medical records and interviews.

### Ethics approval and consent to participate

This study protocol was approved by the Institutional Review Board (IRB) at Korea Advanced Institute of Science and Technology (KAIST), Republic of Korea (approval no.: KH2018-54), and the Severance Hospital, Yonsei University College of Medicine, Seoul, Republic of Korea (approval no.: 4-2012-0859). The study was conducted in accordance with the relevant guidelines and regulations. Written informed consent to participate was obtained from each included patient. All collected tissues were stored in a deep freezer for further analysis.

### Tissue decellularization process

Collected tissues were decellularized using a detergent-based method. The following decellularizing detergent solution was used to remove the cellular components from tissues: 1% (v/v) Triton X-100 (T8787; Sigma-Aldrich, St. Louis, MO, USA) and 0.1% (v/v) ammonium hydroxide (221228; Sigma-Aldrich) in distilled water. Tissue samples were cut into small sections (3 × 3 × 3 mm) and treated with decellularizing solution for >2 h; the solution was replaced at 30-min intervals or when it became opaque. When the tissue became colorless, the resulting patient-derived ECM (pdECM) samples were washed with Dulbecco’s phosphate buffered-saline (Welgene, Gyeongsan, Korea) for 2 days; the solution was replaced at 1-h intervals. Then, the tissue was washed with distilled water, 4 times for 10 min each, to remove residual Dulbecco’s phosphate buffered-saline. Decellularization was performed on an orbital shaker at room temperature, using a speed of 70 rpm. Finally, pdECM samples were lyophilized for 1 day and stored at −20 °C until use.

### pdECM characterization

For hematoxylin and eosin staining, native tissues and decellularized tissues were fixed in 4% paraformaldehyde (Biosesang, Seongnam, Korea) for 1 day and embedded in Paraplast (Leica Biosystems, Wetzlar, Germany); each sample was cut into 10-µm-thick sections. The sectioned samples were stained with hematoxylin and eosin using the standard protocol with slight modification. The DNA content in pdECM samples was quantified using the DNA extraction kit (Bioneer, Daejeon, Korea) in accordance with the manufacturer’s recommendations, and DNA concentrations were measured using a DS-11 Spectrophotometer (DeNovix, Wilmington, DE, USA).

### Proteomic analysis of pdECM with tandem mass tag (TMT) spectrometry

All decellularized gastric tissues were processed using the S-Trap™ mini (ProtiFi, Huntington, NY, USA) [11] to perform protein extraction and digestion, following a slightly modified version of the manufacturer’s instructions. Peptides from each individual and pooled tissues were labeled using the TMT 11-plex (Thermo Fisher Scientific). The TMT-labeled peptides were divided into 20 fractions using a Shimadzu HPLC system. Then, fractions were dried in a SpeedVac vacuum centrifuge and dissolved in 0.1% formic acid for LC-MS/MS analysis. A nano-flow ultra-high-performance liquid chromatography (UHPLC) system (UltiMate 3000 RSLCnano System; Thermo Fisher Scientific) coupled to the Orbitrap Eclipse™ Tribrid™ mass spectrometer (Thermo Fisher Scientific) was used for acquiring UHPLC-MS/MS data. For proteomics analysis, raw files were converted to MS (.ms1) and MS2 (.ms2) files using RawConverter (The Scripps Research Institute, La Jolla, CA, USA). Proteome search and database generation were conducted using IP2 (Integrated Platform for mass spectrometry data analysis, Bruker). Proteome results were analyzed using ProLuCID, DTASelect2, and Census. The database for analysis was generated using the UniProt human proteome database (20,645 entries, updated on January 01, 2020). The following IP2 parameters were used: precursor and fragment mass tolerance, 50 ppm; enzyme, trypsin; miscleavages, ≤ 2; static modifications, 57.0215 Da added at cysteine, 229.1629 Da added at lysine and N-terminal; differential modifications, 15.9949 Da added at methionine; and minimum number of peptides per protein, 2. Pooled spectral files from all 20 fractions were compared with both normal and reversed databases using the same parameters. For peptide validation, the false positive rate was 0.01 of the spectrum level. TMT reporter ion analysis was conducted using Census software, with a mass tolerance of 20 ppm.

Three TMT channels were used as internal references with a pooled common control, which represented pooled peptides of equal amounts from all samples; this approach allowed the assessment of intra- and inter-batch variance, while enhancing quantitative accuracy. The pooled common control was labeled with TMT 130N, 131C, and 131N reagents at a ratio of 0.5:1:2; these reagents served as reference channels. Using the central limit theorem, the log2 ratio of the three reference channels (log2 TMT channels 131N/131C, 131C/130N, and 131N/130N) for all peptides measured in the proteomic analysis was expected to fit a standard Gaussian distribution with near one (131N/131C), near one (131C/130N), and near two (131N/130N), respectively; this method can be used to assess variations in technical replications. We implemented a filtering criterion based on the multidimensional significance offered by Perseus [12]. The Benjamini–Hochberg false discovery rate was used for truncation, with a threshold value of 0.05. Using these criteria, the outlier spectrum was filtered to enhance quantitative accuracy.

Because of differences in sample handling and laboratory environments, there were systematic and sample-specific biases in the quantification of protein abundance. To eliminate these effects, we calculated the median of log2-transformed peptide abundance; column values were subtracted from median values to achieve a common median of 0. Then, we calculated the average of the median values, re-added them to the zero-centered column, and transformed the re-centered value using the y = 2 ^ (x) function (Supplementary Table 1C). For inter-sample intensity normalization, the relative intensity value of each protein was calculated through division of the intensity values of the proteins in each sample by the original intensity value of the R2 column, which was used as the reference for other samples. Then, the final normalized intensity values were calculated through multiplication of the relative intensity value of each protein by the average normalized intensity value of the R2 column. The normalized value was transformed using the y = 2 ^ (x) function. The abundance values were used for further proteome analysis.

### Statistical analysis for TMT proteomics

TMT-based proteomics data were used to perform hierarchical clustering, Principal component analysis (PCA), and differentially expressed protein (DEP) analysis. The difference of RPC of each matrisome categories between NAT and Tumor was determined using Student’s *t* test. For hierarchical clustering, the normalized intensity values were scaled and clustered with the matrisome protein data based on the Euclidean distance in Perseus software [12]. For PCA, only normalized intensity values of matrisome proteins were used. DEPs between the tumor and normal tissues were determined using Welch’s *t*-test with Benjamini–Hochberg correction. DEPs with fold-change > $\sqrt{2}$ and adjusted *p* < 0.01 were selected. Gene set enrichment analysis (GSEA) of DEPs was performed using gene sets provided by Metascape and *p*-values were used to identify enriched genes [13].

### Immunohistochemistry (IHC)

Immunohistochemical studies were carried out on 4-µm formalin-fixed paraffin-embedded (FFPE) tumor tissue slide sections. The slides were deparaffinized in xylene substrate and absolute alcohol, after which they were rehydrated in a decreasing alcohol gradient ending with water. Antigen retrieval was performed by heating the slides in a microwave oven in 10 mM sodium citrate buffer (pH 6.0) for 10 min before blocking endogenous peroxidase activity using 3% hydrogen peroxide in methanol for 30 min. After a brief rinse in TBS, potential nonspecific reactions were blocked by incubating the sections in 5% BSA (HAPLN1) or 10% BSA (COL12A1, THBS2) for 30 min, followed by incubation with primary antibodies either to HAPLN1 (goat antihuman polyclonal Ab, 1:400 dilution, Biotechne, MN, USA), COL12A1 (rabbit antihuman polyclonal Ab, 1:200 dilution, Sigma-Aldrich, MA, USA) or THBS2 (mouse antihuman monoclonal Ab, 1: 1000, Invitrogen, MA, USA) overnight at 4 °C. After washing the slides with TBS, they were incubated for 30 min with the appropriate secondary antibody using Vectastain ABC kit (Vector Laboratories, CA, USA) diluted 1:200 in TBS and detected using DAB solution (Dako, CA, USA). Sections were counterstained with hematoxylin, dehydrated with ethanol in increasing concentrations and mounted with synthetic mountant (Thermo Fisher Scientific, MA, USA) under a coverslip.

### scRNA-Seq and data analysis

We collected public 10×3’ CRC scRNA-seq datasets from Gene Expression Omnibus (GSE166555, GSE188711, GSE132257, GSE132465, GSE144735, GSE178318), PubMed (PMID : 32561858), and Human Cell Atlas, and integrated them with Scanpy v.1.8.2. To integrate these data, we realigned the gene columns of each dataset to the GRCh38 human reference genome using the official Cell Ranger reference v.2020-A. Subsequently, the cells were projected onto batch-corrected Uniform Manifold Approximation Projection (UMAP) space using Harmony [14]. For quality control, we considered cells with <2000 UMI counts and <500 genes detected as empty droplets and excluded them from downstream analysis. The remaining cells were categorized into major cell types (i.e. epithelial, mesenchymal, T cell, B cell, and myeloid) based on representative cell type marker genes. Cells expressing irrelevant cell type markers (e.g. epithelial cells expressing B cell markers) or dominated by a few patients were removed. To annotate each major cell type at higher resolution, we extracted cell type of interest (e.g. mesenchymal cells), projected the cells onto a new batch-free UMAP space using Harmony, identified sub-clusters at varying resolution depending on the cell type, and annotated based on previously reported marker genes and signatures.

To delineate the cell states that shape the intricate tumor microenvironment, we employed Non-negative Matrix Factorization (NMF) analysis on log-normalized and zero-centered scRNA-seq data with negative values converted to zero. Specifically, we first selected major cell type of interest (e.g. mesenchymal cells) and conducted NMF analysis for each tissue using the sklearn.decomposition.NMF in scikit-learn package v.1.0.2 with K value ranging from 5 to 9. This process yielded 35 NMF modules for each tissue. Then, we projected NMF modules to a lower dimension for quantitative assessment and computational efficiency. To achieve this, we max-normalized NMF modules, selected highly variable genes, and filtered out modules with summed NMF weights less than 10–20 or greater than 150–170 depending on cell type. These filtered NMF modules were then projected and clustered in UMAP-space to define cell states with top 50 representative genes. Furthermore, we identified and removed low-quality cell states based on the following criteria: i) those overwhelmed by ribosomal or mitochondrial genes, ii) those derived from a single-study, and iii) those composed of marker genes of irrelevant cell types (e.g. T cell genes in mesenchymal cell states). Finally, we scored all cells with NMF-defined cell states using sc.tl.score to compare the enrichment score between cell types and removed soup-contaminated or doublet states. For instance, if epithelial cell state score was higher in irrelevant cell type (e.g. B cells) than in epithelial cells, we considered it as soup-contaminated or potential doublet state and removed it.

The cellular origins of DEPs were identified using the average expression levels of cell types. Cell type-specific genes were defined using the FindAllMarkers function in the Seurat package; an adjusted *p* < 0.01 was used as a threshold to determine whether the gene expression was cell type-specific. The cell type-specific average expression levels were determined using the AverageExpression function in the Seurat package; the cell type with the highest average expression level was regarded as the cellular origin of the gene. To define the origin of NAT/Tumor enriched matrisome, we used only fibroblasts that were previously annotated with the fibroblast cell type. The gene expression patterns of fibroblasts were normalized and clustered by 1) performing linear dimensional reduction using the RunPCA function in the Seurat package with all matrisome genes regarded as features, 2) using the FindNeighbors function in the Seurat package with the parameter dims = 1:20, 3) using the FindClusters function in the Seurat package with the parameter resolution = 0.5, and 4) using the RunTSNE function in the Seurat package with the parameter dims = 1:20 to plot fibroblasts in the dimensional space. The expression of 39 DEP encoding genes which are mainly expressed in fibroblasts was identified by cell states of fibroblasts.

### Bulk tissue RNA-Seq and bioinformatics analysis

The collected CRC tissues were maintained in TRIzol reagent for bulk tissue RNA-Seq. The indexed cDNA sequencing libraries were prepared from RNA samples using the TruSeq Stranded mRNA LT Sample Prep Kit. Quality control analyses of RNA integrity number and rRNA ratio were performed using the 2200 TapeStation. The indexed libraries were prepared as equimolar pools and sequenced on the NovaSeq 6000 to generate a minimum of 60 million paired-end reads per sample library. The raw Illumina sequence data were demultiplexed and converted to fastq files. Then, the adaptor and low-quality sequences were trimmed. The mRNA sequencing reads were mapped to *Homo sapiens* genome assembly GRCh37 from the Genome Reference Consortium by HISAT2 (version 2.1.0). Mapped reads were assembled with known genes and quantified in terms of read counts and sample normalized values, such as fragments per kilobase of transcript per million mapped reads and transcripts per million mapped reads (TPM), using StringTie (version 2.1.3b).

TCGA-COAD, and TCGA-READ gene expression datasets and a clinical dataset from the TCGAbiolinks package were collected for analyses of CMS-specific gene expression patterns [15]. After the gene expression information had been downloaded from the Illumina platform, the raw counts were converted to normalized TPM values. Clinical information (e.g., the parameters days_to_last_follow_up, death_days_to, and new_tumor_event) was collected and used for analysis of progression-free survival (PFS). In total, 612 tumor samples and 51 normal samples were analyzed. Patients were stratified into two groups based on the expression levels of a specific gene: the high group comprising individuals with expression in the top 25%, and the low group consisting of those with expression in the bottom 25%. Subsequently, a clinical analysis was conducted by comparing the PFS between the high and low expression groups. PFS was calculated using the log-rank test with Kaplan–Meier curve. All statistical tests were two-sided, and statistical significance was defined as *p* < 0.05.

For CMS classification, the CMSclassifier package was used to identify the CMS of collected CRC tissues and TCGA samples [9]. Gene expression values were used after log2-transformation of TPM data and summed to the nearest 0.001. The NearestCMS values and CMS4 probability were calculated using the random forest algorithm. Samples with an ambiguous CMS classification, where the assigned subtype did not constitute a single subtype, were not used for further analysis.

For the single-cell level deconvolution of TCGA bulk RNA-seq of CRC, CIBERSORTx tool was utilized. To create the signature matrix, integrated scRNA-seq data and 33 cell state annotation were used and scRNA-seq data was downsampled by 300 cells per each cell state to make the reference count matrix. Using the signature matrix, normalized TPM values of TCGA-COAD/READ RNA-seq data were used and the cell fraction of each cell states was imputed.

Specific-condition enriched cell state was determined by satisfying two conditions; 1) The median value of cell fraction of the condition is the greatest, 2) *p*-values calculated by Student’s *t* test with any other condition are lower than 0.05.

The expression patterns of specific gene sets in each TCGA sample were evaluated using single-sample GSEA (ssGSEA) [16]. Normalized TPM data of CMS-classified TCGA samples were preprocessed. The ssGSEA scores for gene sets associated with 45 desmoplastic fibroblast matrisome gene markers and 42 PI16+ fibroblast matrisome gene markers were calculated using the ssGSEAprojection package in the GenePattern web-based tool. The calculated scores were log2-transformed and normalized to determine correlations among ssGSEA scores. The normalized score of each gene set for cell-state markers among the CMS was statistically evaluated by pairwise *t*-tests and expressed with compact letter display.

For identification of CMS4-enriched matrisome genes, normalized TPM data of TCGA samples were subjected to GSEA [17, 18]. In total, 68 matrisome markers defined as CMS4-enriched matrisome genes. CMS4-enriched matrisome proteins were defined by comparing the average intensity of the protein from CMS4 versus the other CMS type. If the average intensity of the protein from CMS4 is greater than from the other CMS type, the protein was defined as CMS4-enriched matrisome proteins. The intersection of the CMS4-enriched matrisome genes and the CMS4-enriched matrisome proteins led to the definition of 20 CMS4-enriched matrisome molecules.

## Results

### Quantitative proteomic analysis of decellularized CRC patient-derived tissue

**Fig. 1:**
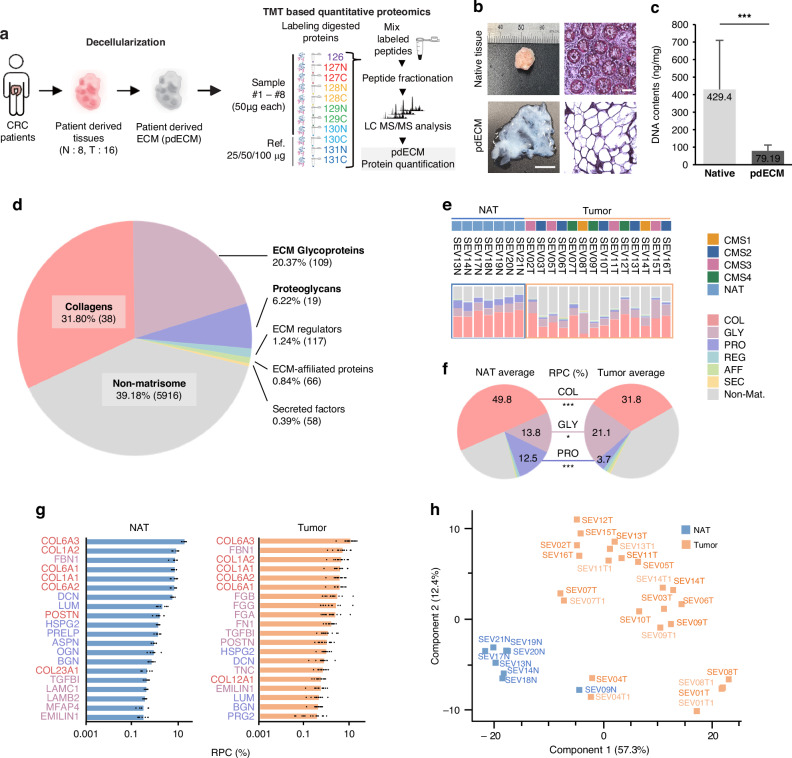
Matrisome-focused proteomic profiles of patient-derived NAT and tumor ECM. **a** Scheme of ECM proteomic analysis of patient-derived tissue. **b** Representative photo and H&E images of native tissue and pdECM. **c** DNA quantification of native tissue and pdECM. (***p < 0.001). **d** Quantitative abundance of matrisome proteins in equally mixed internal reference sample. The number of detected proteins were expressed in the parentheses. **e** Relative percentage composition (RPC) of matrisome proteins among samples, indicated by bar plot. **f** Average RPC of NAT and tumor groups. The RPC values of COL, GLY, PRO are shown and expressed significant difference. *<0.05, ***<0.001. **g** Bar plot shows the most abundant 20 matrisome proteins in NAT and Tumor. RPC of each protein of each sample are shown as dots in the bar plot. **h** PCA plot of all samples. Technical replicates of tumor samples were plotted close to the original samples and are indicated by lighter color text. CRC Colorectal cancer, TMT Tandem mass tag, LC liquid chromatography, MS mass spectrometry, NAT Normal adjacent to tumor, COL Collagens, GLY ECM glycoproteins, PRO Proteoglycans, REG ECM regulators, AFF ECM-affiliated proteins, SEC Secreted factors

To investigate the composition of ECM proteins in CRC, NAT and tumor tissue were acquired surgically from 21 CRC patients. We utilized detergent-based decellularization to enrich ECM proteins and acquired pdECM (Fig. 1a). Supplementary table summarizes the clinical data, tumor stage, location, and consensus molecular subtype (CMS) for each patient. Hematoxylin and eosin stain (H&E) and DNA quantification confirmed the enrichment of ECM proteins (Fig. 1b, c). We confirmed that ECM enrichment process satisfied a substantial loss of nuclei, a reduction in genomic DNA, and the preservation of ECM architecture.

For comparative proteomics analysis of ECM-enriched samples, liquid chromatography-mass spectrometry (LC-MS/MS) analysis on an isobaric tandem mass tag (TMT) was utilized (see Methods for details). In total, we identified 6,323 proteins with no “NA” values in all samples of one set. According to the Human Matrisome Database [19], 407 of these proteins were identified as matrisome proteins (collagens [COLs], ECM glycoproteins [GLYs], and proteoglycans [PROs]) and matrisome-associated proteins [20].

We compared the relative percent composition (RPC) of matrisome proteins found in pdECM samples to those found in native tissue. The RPC of each protein was computed by dividing the intensity of each protein by the sum of the intensities of all detected proteins, and expressed as a percentage. The RPC of each category of matrisome was calculated by adding the RPCs of all proteins corresponding to that category of matrisome. The RPCs of the reference sample (mean value of four internal reference samples of four TMT sets) represent the protein composition of pdECM as shown in Fig. 1d. The total RPC of matrisome proteins was significantly greater in pdECM than in non-decellularized native tissues [21] (Supplementary Fig. 1). The RPCs of the core matrisome, account for 58.67%, which is about sevenfold greater than the RPC in native tissues (8.92%) (Fig. 1d, Supplementary Fig. 1). The RPC of non-matrisome proteins agreed with the RPC measured in other decellularization studies (32–41%) [22, 23]. To identify the linked cellular components of main component protein, a Gene Ontology (GO) analysis of the top 100 proteins with the greatest intensities was identified (Supplementary Fig. 1). ECM-associated proteins were shown to be enriched in pdECM, but nuclear-associated and intracellular proteins were not. In contrast, cytosolic and nuclear proteins were shown to be more abundant in non-decellularized tissue [21] (Supplementary Fig. 1). These observations indicate that our ECM-protein enrichment approach enables detailed identification of matrisome components by LC-MS/MS.

### Quantitative ECM proteomics analysis of pdECM from NAT and tumor tissues

To examine the matrisome components in pdECM of NAT and tumor tissues, we compared their quantitative proteomic profiles. pdECM of NAT and tumor tissue showed significant difference. The RPC of GLYs was significantly increased in tumor tissues (Fig. 1e, f). Also, matrisome-associated proteins and secreted factors were increased in tumor tissues. In contrast, COLs were significantly reduced (from 49.8% to 31.8%). Interestingly, consistent with the global changes in collagens, PROs also decreased significantly from 12.5% to 3.7%. These global change of matrisome contents directly show the dramatic ECM remodeling in tumor tissue.

To examine the main matrisome components in pdECM of NAT and tumor tissues, the top 20 matrisome proteins with the highest average RPC were identified (Fig. 1g). The top 6 most abundant proteins encoded by COL6A1/2/3, COL1A1/2, and FBN1 detected in both NAT and tumor tissues and exhibited a similar trend in both composition and abundance. However, the other top 20 matrisome proteins differed between NAT and tumor tissues, which is consistent with previous studies of the matrisome in CRC tissues [24]. Among the 20 proteins, 13 were highly expressed in both normal and tumor tissues. Three type VI COLs (encoded by COL6A1, COL6A2, and COL6A3) and two type I COLs (encoded by COL1A1 and COL1A2) constituted a significant proportion of the human colon ECM in NAT (55.6%) and tumor (31.8%) tissues. Decorin (DCN) and lumican (LUM), which are involved in the regulation of COL fibril assembly and stability [25, 26], had high abundances in both NAT and tumor tissues; but their levels were much higher in NAT tissue than in tumor tissue. In contrast, GLYs, such as the fibrinogen family (FGA, FGB, and FGG), fibronectin (FN1), transforming growth factor beta-induced protein (TGFβI), and tenascin-C (TNC), had an increased presence in tumor tissues.

Notably, the expression profiles of COLs and PROs were inversely correlated with the levels of the metzincin family of metalloproteinases, including two matrix metalloproteinases (MMPs; MMP9 and MMP14) and two disintegrin and metalloproteinases (ADAMs; ADAM9 and ADAM10) (Supplementary Fig. 2); these metalloproteinases play key roles in ECM remodeling that involve the proteolytic degradation of ECM components [27].

PCA also revealed a difference between the NAT and tumor groups (Fig. 1h). Notably, the PCA results showed not only the different matrisome profile between the NAT and tumor tissue but also their heterogeneity of matrisome profile within the samples. PCA showed greater distances among tumor tissues but not in NAT tissues. Replicate samples were located near each other in the PCA plot, which confirmed the reproducibility of the proteomics analysis. The calculated distance coefficients between the NAT and tumor tissues also indicated that normal tissues are generally similar. In contrast, tumor tissues were generally heterogeneous (Supplementary Fig. 3). Hierarchical clustering with a matrisome profile also indicated that, across multiple patients, all NAT tissues clustered together and demonstrated similar proteomic expression patterns (Supplementary Fig. 4). When we rank the detected proteins according to RPC of each protein of each condition (NAT/Tumor), 81 and 855 protein components covered 90% of RPC in pdECM samples of normal and tumor tissues, respectively (Supplementary Fig. 5). This result also support that the protein components of normal tissues were more uniformly distributed among the samples, compared with the protein components of tumor tissues. Our data suggest that the key ECM components exhibit substantial changes in the amount and composition of ECM in CRC tissues and reveal the ECM heterogeneity which emphasize the necessity of not only the global proteomic analysis but also the tailored ECM-centric analysis.

We note that some samples were excluded for further analysis because of factors that could have affected ECM composition, such as chemotherapy, perforation, or stent insertion (SEV01T: perforation; SEV04T: chemotherapy; SEV09N: stent insertion). The excluded samples showed protein expression patterns distinct from others (Fig. 1h, Supplementary Fig. 4). These results indicate that the ECM composition is also associated with the pathological features of each clinical sample.

### Differentially expressed matrisome proteins in pdECM samples of NAT and tumor tissues

**Fig. 2:**
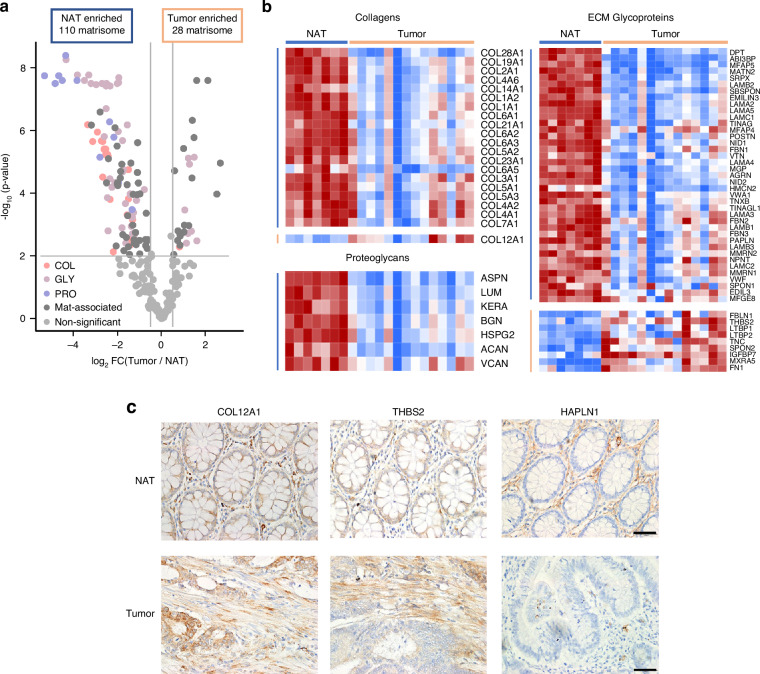
Differentially expressed protein of patient-derived NAT and tumor ECM. **a** Volcano plot of matrisome proteins. Gray lines show thresholds for DEPs with log2 (fold change) >0.5 and adjusted *p* < 0.01. The 28 tumor-enriched DEPs are shown on the right side and 110 NAT-enriched DEPs are shown on the left side. **b** Heatmap of all DEPs included in the core matrisome category. **c** Immunohistochemical images of COL12A1-, THBS2-, and HAPLN1-stained NAT and tumor tissues. Scale bar: 50 μm.

To determine compositional changes in the ECM proteins, we compared the matrisome of NAT and tumor tissues by DEP analysis. For each protein, we calculated the fold-change between NAT and tumor tissues, along with adjusted *p*-values according to Welch’s *t*-test. Volcano plots in Fig. 2a show matrisome DEPs; 110 and 28 matrisome proteins were enriched in pdECM samples of NAT and tumor tissues, respectively (Detailed DEP list in Supplementary Table). The heatmap of core matrisome DEP proteins showed significantly upregulated proteins in NAT and tumor tissues (Fig. 2b).

Among the tumor-enriched DEPs within the core matrisome, nearly all are included in the GLY group, with α1 chain of collagen XII (COL12A1) being the only DEP not part of the GLY group. (Fig. 2b). *COL12A1* has been reported as a novel stromal marker with robust expression in the desmoplastic stroma of CRC tissues [28]. Among the tumor-enriched GLYs, matrix-remodeling associated protein 5 (MXRA5) had the greatest statistical significance (*p* = 7.13 ×10^−6^). Multiple COLs, GLYs, and PROs were abundantly present in NAT tissues. In particular, PROs in the small leucine repeat proteoglycans (SLRPs) family (e.g., decorin [*DCN*], lumican [*LUM*], asporin [*ASPN*], and osteoglycan [*OGN*]) were most significantly enriched in NAT tissue ECM. The upregulation of proteinases (i.e., MMPs and ADAMTS) in tumor tissues (Supplementary Fig. 2) supports that proteases digestion could induce the depletion of extracellular SLRPs under pathophysiological conditions [29]. Given that the SLRPs regulate COL fibril organization and stability [25], SLRP depletion may cause ECM dysfunction by interfering with COL network stability and accelerating COL degradation in CRCs. NAT-/tumor-enriched DEPs were validated with IHC of tissue section. COL12A1 and THBS2 showed enriched expression in tumor tissue section and hyaluronan and proteoglycan link protein 1 (HAPLN1) showed enriched expression in NAT tissue section (Fig. 2c).

### Stroma-focused single-cell analysis of integrated single cell atlas of CRC

In the proteomic analysis, it was observed that the ECM of NAT differs from that of tumor tissue, with the latter exhibiting greater heterogeneity. We hypothesized that this heterogeneity arises from the stromal cells such as fibroblasts, the primary cell type depositing and degrading the ECM, which themselves also display heterogeneity. We conducted scRNA-seq analysis and developed cell-state-annotated scRNA-seq reference from the integrated public large-scale data to directly demonstrating these phenomena. Additionally, to further associate the heterogeneity of the ECM and the stromal components with molecular subtypes, we employed the deconvolution of bulk sequencing data from TCGA data and the scRNA-seq reference, indirectly demonstrating the remodeled stroma across the entire CRC tissue.

**Fig. 3:**
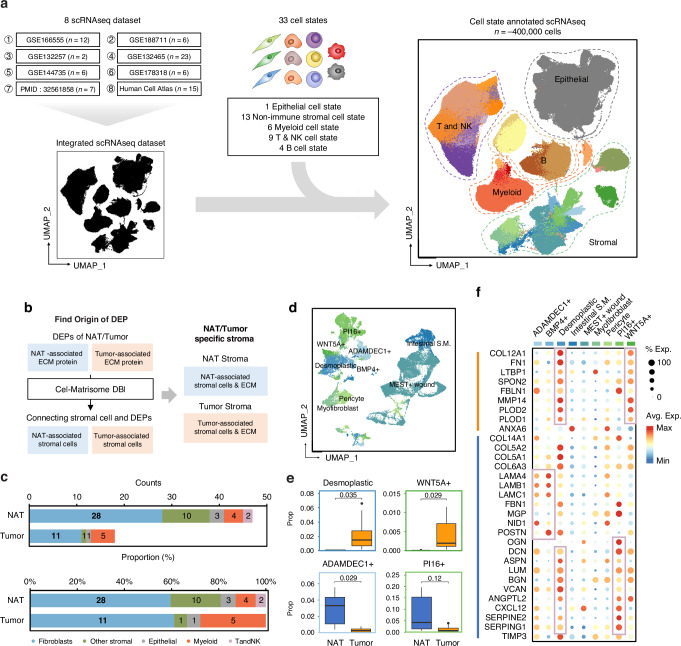
Stroma focused analysis of integrated single cell atlas of colorectal cancer tissue. **a** Integrated single cell atlas of colorectal cancer and NAT colon tissue. 8 scRNA-seq dataset were integrated and cell were annotated with 5 broad cell types and 33 state-based cell types (Methods in detail). **b** Scheme of identifying NAT/Tumor specific stroma. The NAT/tumor specific stroma could be identified by analyzing DEPs between NAT/tumor proteomic profile and the origin cells of defined DEPs. **c** Cellular origin of DEPs. The cellular origin of DEP was annotated with 5 major cell types. **d** UMAP plot of 9 states of fibroblast. **e** Distribution of NAT/tumor enriched fibroblast types in each dataset. The proportion of desmoplastic fibroblast and WNT5A+ inflammatory fibroblast were enriched in tumor dataset, and ADAMDEC1+ fibroblast and PI16+ fibroblast were enriched in NAT dataset. *p*-values were calculated using Wilcoxon test. **f** The single-cell level expression of NAT/tumor enriched matrisome from DEP analysis. The NAT enriched matrisome were mainly derived from ADAMDEC1+ fibroblast, BMP4+ fibroblast, Desmoplastic fibroblast, PI16+ fibroblast. The tumor enriched matrisome were mainly derived from desmoplastic fibroblast, Wnt5A+ inflammatory fibroblast.

To elucidate the primary cellular component driving ECM remodeling, scRNA-seq data could be utilized to connect the matrisome gene. We integrated 8 public scRNA-seq data to construct a single cell atlas of CRC (Fig. 3a). About 400,000 single cells were analyzed and firstly divided with 5 major categories (epithelial, myeloid, T&NK, B, non-immune stromal cell). Then, the cells were further annotated with 33 cell states according to their expression. 33 cell states were composed of 1 epithelial cell state, 13 non-immune stromal cell states, 6 myeloid cell states, 9 T&NK cell states, 4 B cell states.

The non-immune stromal cells dominated the cell states (13 of 33 cell states). These included *ADAMDEC1*^+^ fibroblast (*ADAMDEC1*, *APOE*), *BMP4*^+^ fibroblast (*CXCL14*, *F3*), desmoplastic fibroblast (*CTHRC1*, *SULF1*), intestinal smooth muscle (*MYH11*, *ACTG2*), *MEST*^+^ wound fibroblast (*MEST*, *STMN1*), myofibroblast (*ADIRF*, *PLN*), pericyte (RGS5, *NDUFA4L2*), *PI16*^+^ fibroblast (*CFD*, *MGP*), *WNT5A*^+^ inflammatory fibroblast (*MMP1*, *CXCL8*), enteric glial cell (PLP1, S100B), endothelial cell (*PLVAP*, *PECAM1*), lymphatic endothelial (*CCL21*, *TFF3*) and low-quality cells (Supplementary Fig. 6, upper left). 6 myeloid cell states (*TYROBP*, *FCER1G, LYZ*) consist of DC1 (*CLEC9A*, *DNASE1L3*), DC2 (*CD1C*, *CLEC10A*), LAMP3 + DC (*CCR7*, *CCL19*), macrophage (*S100A9*, *CCL3*), mast cell (*TPSAB1*, *TPSB2*), pDC (*GZMB*, *JCHAIN*) (Supplementary Fig. 6, upper right). 9 T&NK cell states consist of *CD16*^+^ NK cell (*GNLY*, *FGFBP2*), *CD160*^+^ intraepithelial lymphocyte (*TRDC*, *HOPX*), *CD4*^+^ T(*IL7R*, *CCR7*), *CD8*^+^ T (*NKG7*, *GZMK*), ILC3 (*AREG*, *TYROBP*), Tfh (*CXCL13*, *NR3C1*), Th17(*KLRB1*, *CCL20*), Treg (*TNFRSF4*, *IL2RA*), *XCL1*^+^ NK (*TYROBP*, *GNLY*) (Supplementary Fig. 6, bottom left). 4 B cell states consist of general B cell (*HLA-DRA*, *HLA-DPB1*), Germinal center B cell (*TCL1A*, *MARCKSL1*), IgA plasma *(IGHA2*, *IGHA1*), IgG plasma (*IGHG1*, *IGHG3*) (Supplementary Fig. 6, bottom right).

The distribution of cell states except epithelial cell state was identified along the dataset (Supplementary Figure 6). Some cell states were differently enriched between NAT and tumor tissues. For example, *ADAMDEC1*^+^ fibroblasts, *PI16*^+^ fibroblasts, and IgA plasma appear to be enriched in NAT tissues, in contrast to desmoplastic fibroblasts, macrophages, Treg cells, and IgG plasma, which exhibit a higher proportion in tumor tissues. These findings align with the previous research [30–32].

Upon comparing the expressions of genes corresponding to the matrisome across various cell states, it is conceivable that specific matrisome genes may serve as markers for each cell state. Establishing such associations could be facilitated through the creation of a cell-matrisome database (DB), thereby elucidating the cell states accountable for particular matrisome expressions.

Supplementary Fig. 6 summarized the matrisome markers associated with each cell state. In the case of desmoplastic fibroblasts, distinctive expression profiles were observed, featuring COL family members such as *COL type 8/10/11/12, MMP11/14*, and GLYs such as thrombospondin 2 (*THBS2*) and matrix remodeling associated 5 (*MXRA5*). Notably, the expression of genes serving as markers for desmoplastic fibroblasts exhibited some degree of elevation in *WNT5A*^+^ inflammatory fibroblasts, including a distinctive upregulation of the C-X-C motif chemokine ligand (*CXCL*) family, particularly *CXCL 1/3/5/6/8*. Similarly, *PI16*^+^ fibroblasts exhibited unique expression patterns, characterized by the specific expression of PROs like *OGN*, *LUM*, prolargin (*PRELP*), and markers such as COL type 14 (*COL14A1*), dermatopontin (*DPT*), and ABI family member 3 binding protein (*ABI3BP*), previously identified as significantly enriched in NAT tissues. While matrisome genes uniquely expressed by cell types other than fibroblasts were relatively sparse, discernible patterns were evident. For instance, endothelial cells exhibited the expression of matrisome genes such as von Willebrand factor (*VWF*) and EGF like domain multiple 7 (*EGFL7*), whereas macrophages demonstrated elevated expression of S100 calcium binding protein *(S100) A8/9* and complement component 1q (C1Q) A/B/C genes.

Analysis of the C-C motif chemokine ligand (CCL) family genes revealed diverse expression patterns across subtypes and cell states. *ADAMDEC1*^+^ fibroblasts exhibited elevated expression of *CCL2/8/11/13*, lymphatic endothelial cells expressed *CCL21*, macrophages demonstrated *CCL3* expression, *LAMP3*^+^ dendritic cells displayed *CCL19/22*, and *CD16*^+^ NK cells exhibited increased expression of *CCL4/5* genes, thus identifying these genes as part of their matrisome. The establishment of a cell-matrisome database, linking cell states with matrisome genes, provides a promising avenue for predicting cell states associated with distinctive stromal features.

### Identification of differential cellular origin expressing NAT-associated and tumor-associated matrisome proteins

To comprehensively understand the differences between NAT stroma and tumor stroma, we employed the DEPs and cell-matrisome DB to define cells associated with NAT and tumor stroma (Fig. 3b). Initially, we investigated the cell types majorly expressing genes encoding DEPs. Out of a total of 110 NAT-enriched proteins and 28 tumor-enriched proteins, 47 and 18 genes, respectively, were annotated as specific cell type markers in the cell-matrisome DB. Notably, 60% of these genes, constituting 28 and 11 genes for NAT and tumor, respectively, were confirmed as fibroblast-derived genes (Fig. 3c). A detailed list of annotations is provided in the Supplementary table.

Before annotating 39 fibroblast-derived matrisome genes based on their state, we conducted an analysis of fibroblast distribution across different states in scRNA-seq data (Fig. 3d, e). Proportions of single cells annotated to each cell state were calculated in each dataset, revealing significant variations in fibroblast distribution across states. In tumor, desmoplastic fibroblasts and *WNT5A*^+^ inflammatory fibroblasts were notably enriched. In contrast, in NAT, *ADAMDEC1*^+^ expressing fibroblasts exhibited remarkable enrichment. Although not statistically significant, a high fraction of *PI16*^+^ expressing fibroblasts was also observed in NAT.

Upon examining the expression of the 39 NAT/tumor-enriched matrisome genes across different cell states, 22 and 9 genes exhibited specific expression patterns. Genes encoding NAT-enriched matrisome proteins were prominently expressed in *ADAMDEC1*^+^, *BMP4*^+^, and *PI16*^+^ expressing fibroblasts. On the other hand, genes encoding tumor-enriched matrisome proteins were predominantly expressed in desmoplastic fibroblasts and *WNT5A*^+^ inflammatory fibroblasts (Fig. 3f). Noteworthy is the observation that certain matrisome genes, such as PROs primarily expressed in *PI16*^+^ fibroblasts, also exhibited substantial expression in desmoplastic fibroblasts, suggesting phenotypic changes in resident fibroblasts, like *PI16*^+^ fibroblasts, transitioning into desmoplastic fibroblasts within the tumor microenvironment.

### Stroma deconvolution of colorectal cancer tissue according to molecular subtype

**Fig. 4:**
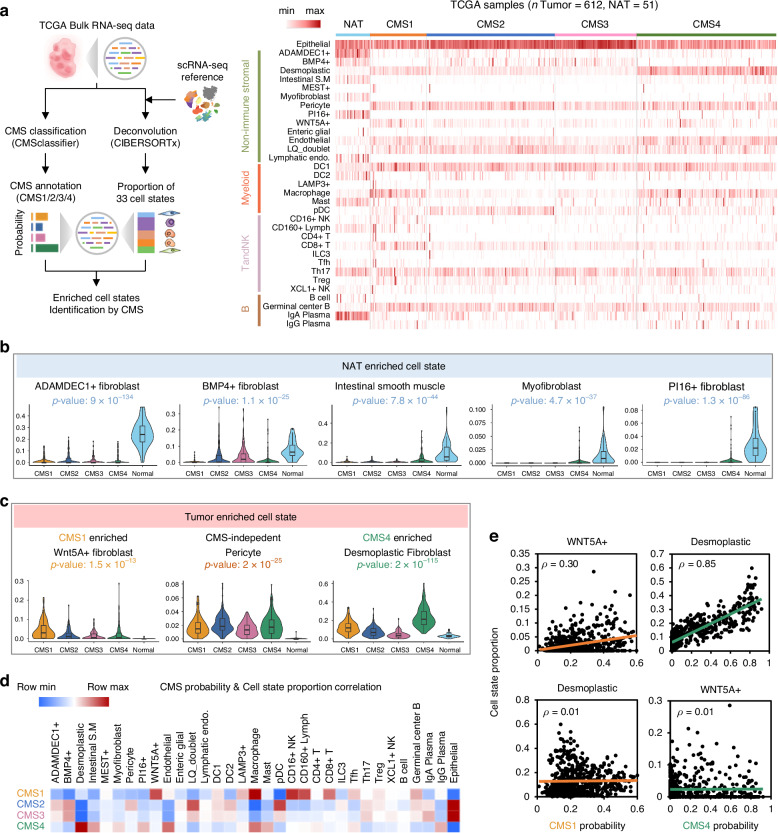
Single-cell level deconvolution of colorectal cancer tissue. **a** The bulk transcriptomic data of TCGA samples were deconvoluted with 33 cell states by CIBERSORTX analysis tool and annotated with consensus molecular subtype (CMS) by CMSclassifier. Normalized cell fraction values of each cell type with CMS annotation were expressed in the heatmap. Violin plot of **b** NAT enriched cell types and **c** tumor enriched cell types. *p*-values were calculated using one-way ANOVA. **d** Heatmap of correlation between CMS probability and proportion of each cell state. The coefficients of Pearson correlation were calculated with each cell state and probability value of each CMS. **e** Dot plot of CMS probability and the proportion of each cell state. Each dot represents each sample in TCGA samples.

The identification of NAT and tumor stroma components, encompassing ECM and stromal cells, was achieved through proteomic analysis and scRNA-seq. This identification process can be further validated using bulk RNA-seq data and deconvolution tools such as CIBERSORTX [33]. Deconvolution enables the assessment of the distribution of stromal cells based on their states within each tissue. Using the single-cell gene expression data of 33 cell states, we generated a signature matrix for deconvolution and applied it to TCGA bulk expression data from various NAT/CRC samples (Fig. 4a). Also, the CMS annotation of samples was performed with CMSclassifier R package [9].

The deconvolution results revealed significant differences in distribution of cell state between NAT and tumor samples. Notably, the cell states identified as NAT-enriched in single-cell sequencing, such as *ADAMDEC1*^+^ fibroblast, *PI16*^+^ fibroblast, and IgA plasma, exhibited consistent trends in bulk sequencing deconvolution. Similarly, cell states identified as tumor-enriched in single-cell sequencing, including desmoplastic fibroblast, macrophage, Treg, and IgG plasma, were corroborated as tumor-enriched in bulk sequencing deconvolution.

Among the nine cell states associated with fibroblasts, five (*ADAMDEC1*^+^ fibroblast, *BMP4*^+^ fibroblast, intestinal smooth muscle, myofibroblast, *PI16*^+^ fibroblast) were identified as NAT-enriched, and three (*WNT5A*^+^ fibroblast, pericyte, desmoplastic fibroblast) were identified as tumor-enriched.

Interestingly, tumor-enriched fibroblast cell states exhibited subtype-specific variations across the molecular subtypes (Fig. 4b, c). CMS1 showed a relatively higher proportion of *WNT5A*^+^ fibroblasts compared to other CMS types, while CMS4 exhibited a higher proportion of desmoplastic fibroblasts (Fig. 4c). These trends in stromal cell states could serve as valuable indicators for discerning molecular characteristics of TME. Analyzing the correlation between CMS probability and cell state proportion for all cell states, CMS2 and CMS3 exhibited the highest correlation with epithelial cells, while CMS1 showed high correlations with macrophages, *CD16*^+^ NK cells, and *WNT5A*^+^ inflammatory CAFs. CMS4 demonstrated a strong correlation with desmoplastic fibroblasts and endothelial cells (Fig. 4d). The weak correlations between CMS1 and desmoplastic fibroblasts and CMS4 with *WNT5A*^+^ inflammatory fibroblasts underscore the heterogeneity within fibroblasts across different molecular subtypes, emphasizing distinct definitions of relevant fibroblasts for each molecular subtype of tissue (Fig. 4e).

### CMS4-specific matrisome markers and clinical relevance

CMS4 group is characterized by extensive stromal infiltration (mostly activated fibroblasts) and ECM organization [9]. Recent studies have demonstrated that CAFs in CRCs are composed of distinct fibroblast populations and significantly enriched in the CMS4 subtype compared with the other subtypes [8]. Therefore, we sought to compare ECM features between the CMS4 subtype and other subtypes. Through deconvolution, the cell types enriched in CMS4 were explored, revealing a notably higher proportion of desmoplastic fibroblasts. In addition, the proteoglycan category predominantly expressed in desmoplastic fibroblasts was also found to be expressed in PI16+ fibroblasts, indicating a potential association between *PI16*^+^ fibroblasts and CMS4-specific stroma.

**Fig. 5:**
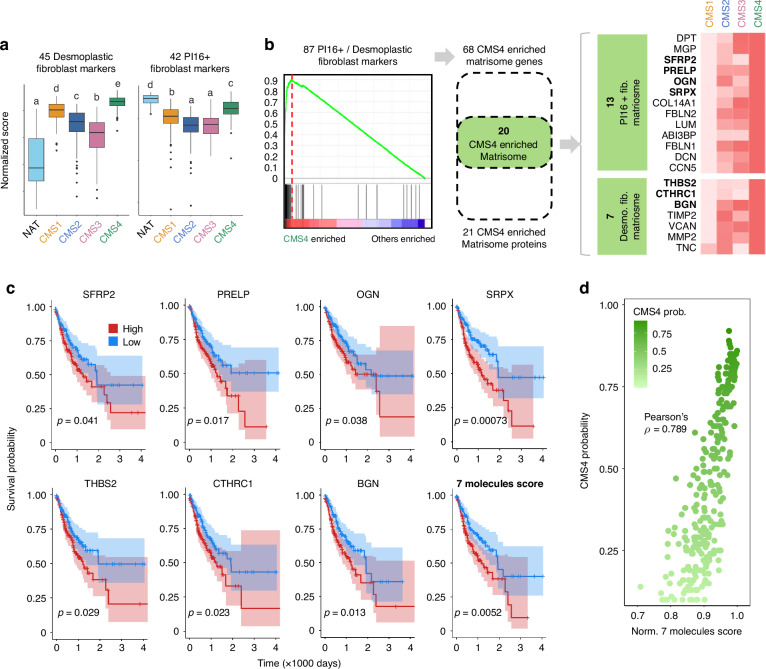
CMS4 subtype enrich PI16+/desmoplastic fibroblast matrisome and predict poor prognosis. **a** Boxplot of normalized score of TCGA samples with the expression of desmoplastic fibroblast markers and PI16+ fibroblast markers. Statistical significance expressed by alphabet letter. Different letters indicate statistically significant differences. (*p*-value < 0.05). **b** 20 CMS4 enriched matrisome were defined with the intersection of gene markers and protein markers. CMS4 enriched matrisome genes were identified with gene set enrichment analysis (GSEA) of CMS4 sample vs others. CMS4-enriched matrisome proteins were identified as proteins with the highest average intensity in CMS4 compared to other CMS types. **c** Progression-free-survival (PFS) analysis of TCGA patients with the molecules that have clinical significance (*p*-value < 0.05). 7 molecules, both individually and simultaneously, predicted poor prognosis. Confidence intervals are represented by the colored areas. d) Dot plot of CMS4 probability and normalized 7 molecules score. The higher normalized 7 molecules score predicted the higher CMS4 probability. The coefficient of Pearson correlation was calculated.

Subsequent ssGSEA on TCGA RNA-seq datasets evaluated desmoplastic fibroblast and PI16+ fibroblast scores across tissues using 45 and 42 marker genes, respectively (Fig. 5a). The desmoplastic fibroblast score exhibited the highest distribution in CMS4, while the *PI16*^+^ fibroblast score, although lower than in NAT tissues, displayed the highest distribution among CMS types. These results indicate elevated expression of desmoplastic fibroblast and *PI16*^+^ fibroblast markers in CMS4 tissues.

Further investigation using GSEA identified 68 marker genes that were significantly enriched in CMS4 out of a total of 87 desmoplastic fibroblast and *PI16*^+^ fibroblast markers (Fig. 5b). Leveraging the ECM proteomics data, 21 proteins highly expressed in CMS4 tissues were identified. The intersection of the 68 CMS4-enriched matrisome genes and the 21 CMS4-enriched matrisome proteins led to the definition of 20 CMS4-enriched matrisome molecules. 13 molecules of these matrisome molecules (DPT, matrix Gla protein [MGP], secreted frizzled-related protein2 [SFRP2], PRELP, OGN, sushi repeat containing protein X-linked [SRPX], COL14A1, fibulin 2 [FBLN2], LUM, ABI3BP, FBLN1, DCN, cellular communication network factor 5 [CCN5]) were associated with PI16+ fibroblasts, while 7 molecules (THBS2, collagen triple helix repeat containing 1 [CTHRC1], biglycan [BGN], TIMP metallopeptidase inhibitor 2 [TIMP2], versican [VCAN], MMP2, TNC) were linked to desmoplastic fibroblasts (Fig. 5b).

Since the CMS4 is widely recognized as the most malignant and prognostically unfavorable CMS type, it is important to refine the molecular marker based on clinical significance. To do this, we performed survival analysis for each marker. Among the 20 CMS4-specific matrisome molecules, 7 showed associations with PFS (Fig. 5c). The 7-gene signature score also predicted a poor prognosis, with reduced overall survival and PFS. We also found significant correlations between the expression levels of the 7 genes and CMS4 probability (Fig. 5c, d). When the normalized expression score of the 7 genes was <0.9, most samples were regarded as the CMS4 subtype (Fig. 5d). All 7 genes showed predominant expression in fibroblasts and were highly enriched in CMS4. Thus, the 7 clinically significant CMS4-specific matrisome genes may be used to infer the fibroblast population in the TME and to discriminate between malignant CMS4 and other subtypes. Our results indicate that the activation patterns of the 7 ECM genes are associated with the malignant stroma of CRC, particularly in the CMS4 subtype. These genes may be used to determine the CMS4-specific ECM components predicting a poor prognosis.

## Discussion

Comprehensive understanding of ECM remodeling in TME is essential for developing antitumor treatments and diagnostic biomarkers. The ECM has been used as a diagnostic and prognostic signature of metastatic potential in CRC [34]. In this work, using quantitative TMT proteomics and ECM enrichment with decellularization, we successfully identified the global ECM proteins in normal and tumorous CRC tissues. We detected nearly 400 ECM proteins in each sample, accounting for 60% of the overall protein intensity. Despite the analytical challenges posed by the particular biochemical properties of extracellular proteins to proteomic techniques, our ECM-enriched approach enabled more sensitive and accurate identification at the proteome level.

Our results showed that CRC tumor tissues exhibit inter-patient heterogeneity in terms of ECM components, whereas histologically normal tissue adjacent to the tumor tissue exhibits ECM protein homogeneity. There is extensive evidence of interpatient heterogeneity based on cell-centered analysis [9], but this heterogeneity has not been reported in the acellular level in TME. Based on a quantitative comparison between tumor and NAT ECM, we observed a substantial loss of PROs (i.e., DCN, OGN, LUM, and HAPLN1). These proteins are well-known for maintaining ECM integrity and structural stability by forming an organized and insoluble structure [35]. The loss of PROs may result in abnormal and immature self-assembly of tumorous ECM proteins during tumor progression. In turn, such alteration could make ECM components more susceptible to degradation and delayed modification [25, 26]. Furthermore, some PROs (e.g., DCN) are key cytokine and growth factor reservoirs that modulate signals transmitted by epidermal growth factor receptors, insulin-like growth factor receptor, and TGF-β [36]. Accordingly, the depletion of tumor-suppressive PROs may be associated with the oncogenic signaling pathway during CRC pathogenesis [37].

In addition to PROs, collagens were significantly depleted in tumor ECM. Although the population of myofibroblasts that can produce collagens is increased in the CRC microenvironment [8], the abundance of collagen proteins is decreased. Disease progression leads to altered collagens [38]; dysfunctional collagen turnover may result in homeostasis failure. Although collagen is continually undergoing deposition and degradation [39], the proteolysis alterations caused by increased levels of MMPs lead to increased matrix turnover and proteolytic degradation of existing collagens [40]. Thus, the normal collagen matrix could be decreased and concurrently replaced by tumor-specific types of collagens (i.e., COL12A1 and COL11A1) in tumor tissues. Our findings are consistent with a previous report suggesting that collagen degradation is a common feature of tumorigenic ECM remodeling in CRC [41]. In addition to the protease-dependent degradation of ECM proteins, the depletion of the normal colon mucosa fibroblasts during tumor progression may cause a severe lack of normal ECM proteins in tumor tissues [42].

A recent study of CRC revealed a functionally distinct subclass of cancer-associated fibroblasts (CAFs) based on reference component analysis of single-cell transcriptomes, suggesting that CAFs comprise a diverse cell population of multiple subtypes [43]. In this regard, our finding on ECM-related fibroblast feature highlighted the functional heterogeneity of CAFs. By integrating scRNA-seq datasets from CRC tissues, we found that the tumor-enriched proteins were predominantly associated with desmoplastic fibroblasts and *WNT5A*^+^ inflammatory fibroblasts. In contrast, PI16+ fibroblasts and *ADAMDEC1*^+^ fibroblasts were associated with normal/NAT.

Two prominent fibroblasts dominantly present in the NAT tissues was PI16+ fibroblasts and *ADAMDEC1*^+^ fibroblasts, as also mentioned in the previous references [30]. Among these, *ADAMDEC1*^+^ fibroblasts are characterized by expressing secreted factors such as *CCL2/11/13*, *CXCl14*, while *PI16*^+^ fibroblasts, on the other hand, can be viewed as fibroblasts associated with ECM deposition and remodeling, expressing not only *COL14A1* but also PROs like *DCN*, *LUM*, *PRELP*, *OGN*. The distinctive features of these tissue-resident fibroblasts can be linked to their molecular phenotype of CRC tissue.

CMS4, known for its prominent cancer cell stroma infiltration, is well-established as a subtype that activates resident fibroblasts, leading to the formation of activated CAFs [9, 44, 45]. In CMS4, *ACTA2*^+^ fibroblasts are commonly expressed at higher levels compared to other types [46], suggesting a correspondence with desmoplastic fibroblast that predominantly express *ACTA2*. Desmoplastic fibroblasts, known for expressing genes associated with ECM remodeling, collagen deposition, and MMPs, may likely derive from *PI16*^+^ resident fibroblasts. The common expression of various ECM-related genes, such as *LUM*, *BGN*, *THBS2*, and *FBLN2*, between *PI16*^+^ fibroblasts and desmoplastic fibroblasts supports this proposition.

On another note, *WNT5A*^+^ fibroblast exhibit lower expression of *ACTA2* compared to desmoplastic fibroblasts. Simultaneously, they share some markers with desmoplastic fibroblasts while additionally expressing immune-related cytokines more prominently than desmoplastic fibroblasts. Notably, this cell type is prevalent in CMS1, suggesting a significant representation of high immune activation and immune evasion pathways in CMS1. As an illustrative example, the CXCL family members, primarily expressed in *WNT5A*^+^ inflammatory fibroblasts, play a crucial role in inflammation and immune regulation. It is reported that one of the CXCL family member, *CXCL5* is associated with immune evasion effects [47]. Also, only *WNT5A*^+^ inflammatory fibroblast mainly expressing *IL6*, which could make immune evasion by interacting immune checkpoint proteins such as PD-L1 and TIGIT [48, 49]. As demonstrated, fibroblasts, rather than immune cells, play a role in shaping the immune microenvironment, exhibiting distinct molecular phenotypes according to CMS. This observation, confirmed through ECM-centric and cell state analyses, contributes to a comprehensive understanding of immunotherapy.

Lastly, we identified specific subsets of ECM molecules associated mesenchymal signature of CRCs. We have shown that the 68 fibroblast-derived matrisome gene markers can be used to distinguish CMS4 from other subtypes and 20 matrisome marker that showed concordant proteomic pattern were identified as CMS4-enriched matrisome. Importantly, our findings are in agreement with the results of a recent study, in which the distinctive transcriptional features of the mesenchymal subtype were ascribed to the stromal contribution linked to the CAF abundance [10]. 7 matrisome markers suggested in the present study were associated with overall survival, consistent with previous findings that the expression levels of CAF genes were increased in CRC patients with poor prognoses [10].

Indeed, in low-risk stage II CRC patient without adjuvant chemotherapy, those with CMS4 subtype had worse survival rates without relapse compared to other subtypes [50]. Therefore, it is crucial to distinguish patients with the mesenchymal CMS4 subtype from others. In this context, a comprehensive understanding of the subtype-specific TME can facilitate the development of subtype-specific diagnosis and stromal-targeted treatment strategies for improving the survival of the CMS4 subtype in CRC.

This study has a few limitations that could be addressed in future research. First, due to the limited availability of colorectal cancer tissue obtained through surgery within a restricted timeframe, the number of possible samples with supplementing both transcriptomic profiles for CMS typing and ECM proteomic profiles was limited. As a result, the number of tissues corresponding to the targeted CMS types only met the minimum statistical requirements. To mitigate this, we performed deconvolution using TCGA bulk RNA-seq data, which provided a larger sample size. Additionally, while we utilized gene expression data from the TCGA database for survival analysis, the clinical relevance of the identified molecules could be further strengthened by linking the expression of these proteins with CMS subtypes and survival rates using tumor microarrays from our own archives. This could provide a more direct validation of the molecules’ clinical significance. Finally, although we defined specific fibroblasts as being enriched in particular stromal subtypes based on gene expression data, the precise manner in which these fibroblasts are situated within the stroma remains unclear. Employing techniques such as multiplex IHC, which can simultaneously analyze ECM and fibroblast markers, allow us to determine whether the target ECM and fibroblasts are correlated and to understand their spatial relationship. This could lead to the development of more effective strategies for therapies targeting specific stromal components.

Conclusively, our findings illuminate the understanding of unprecedented molecular and histopathological feature of CRC. Our integration of ECM-centered proteomics and stroma-focused transcriptomics data promises to offer a truly comprehensive perspective on the molecular landscape of CRC.

## Supplementary information

Supplementary Figures

Supplementary table legend

Supplementary table

## Data Availability

The data supporting the findings of this study are available within the paper and its Supplementary Information. All data is available on request from the authors. All MS data and search results files were deposited in ProteomeXchange Consortium via the MassIVE partner repository with the identifier PXD037824 for ProteomeXchange and MassIVE MSV000090604 for MassIVE.
