# Correction: Differential cellular origins of the extracellular matrix of tumor and normal tissues according to colorectal cancer subtypes

**DOI:** 10.1038/s41416-025-02988-5

**Published:** 2025-04-11

**Authors:** Hyun Jin Lee, Sang Woo Park, Jun Hyeong Lee, Shin Young Chang, Sang Mi Oh, Siwon Mun, Junho Kang, Jong-Eun Park, Jung Kyoon Choi, Tae Il Kim, Jin Young Kim, Pilnam Kim

**Affiliations:** 1 Department of Bio and Brain Engineering, KAIST, Daejeon, 34141 Republic of Korea; 2 Research Center for Bioconvergence Analysis, Korea Basic Science Institute, Ochang, 28119 Republic of Korea; 3 Department of Internal Medicine, Institute of Gastroenterology, Brain Korea 21 Project for Medical Science, Severance Hospital, Yonsei University College of Medicine, Seoul, 03722 Republic of Korea; 4 Graduate School of Medical Science and Engineering, KAIST, Daejeon, Republic of Korea; 5 SCL-KAIST Institute of Translational Research, KAIST, Daejeon, Republic of Korea

**Keywords:** Cancer microenvironment, Colorectal cancer, Tumour heterogeneity, Proteome informatics

Correction to: *British Journal of Cancer* 10.1038/s41416-025-02964-z, published online 03 March 2025

In this article the statement in the Funding information section was initially incomplete; it originally read: Open Access funding enabled and organized by KAIST.

The complete Funding information section is as follows:

Korea Health Industry Development Institute (KHIDI) - HA22C0050; National Research Foundation of Korea (NRF) - NRF-2022R1A4A502813112; National Research Foundation of Korea (NRF) - NRF-RS-2024-00338828; National Research Foundation of Korea (NRF) - NRF2019R1A2C2084142; Korea Health Industry Development Institute (KHIDI) - HI14C1324.

The original article has been updated.

