# Supplementary material for: Differential cellular origins of the extracellular matrix of tumor and normal tissues according to colorectal cancer subtypes
Source: Br J Cancer. 2025 Mar 3;132(9):770–82. doi: 10.1038/s41416-025-02964-z (PMC12041468; doi:10.1038/s41416-025-02964-z)
Supplement: Supplementary file 1 — Supplementary Figures [file 41416_2025_2964_MOESM1_ESM.pdf]

Supple. Fig. 1

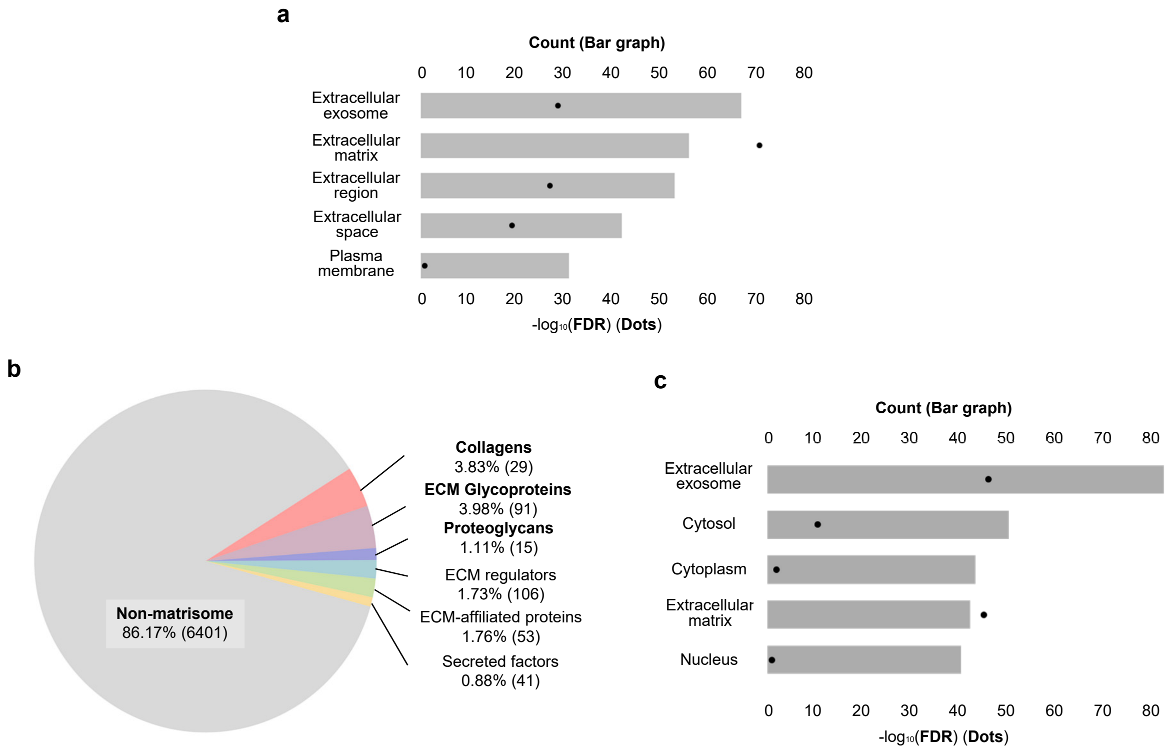

Supple. Fig. 2

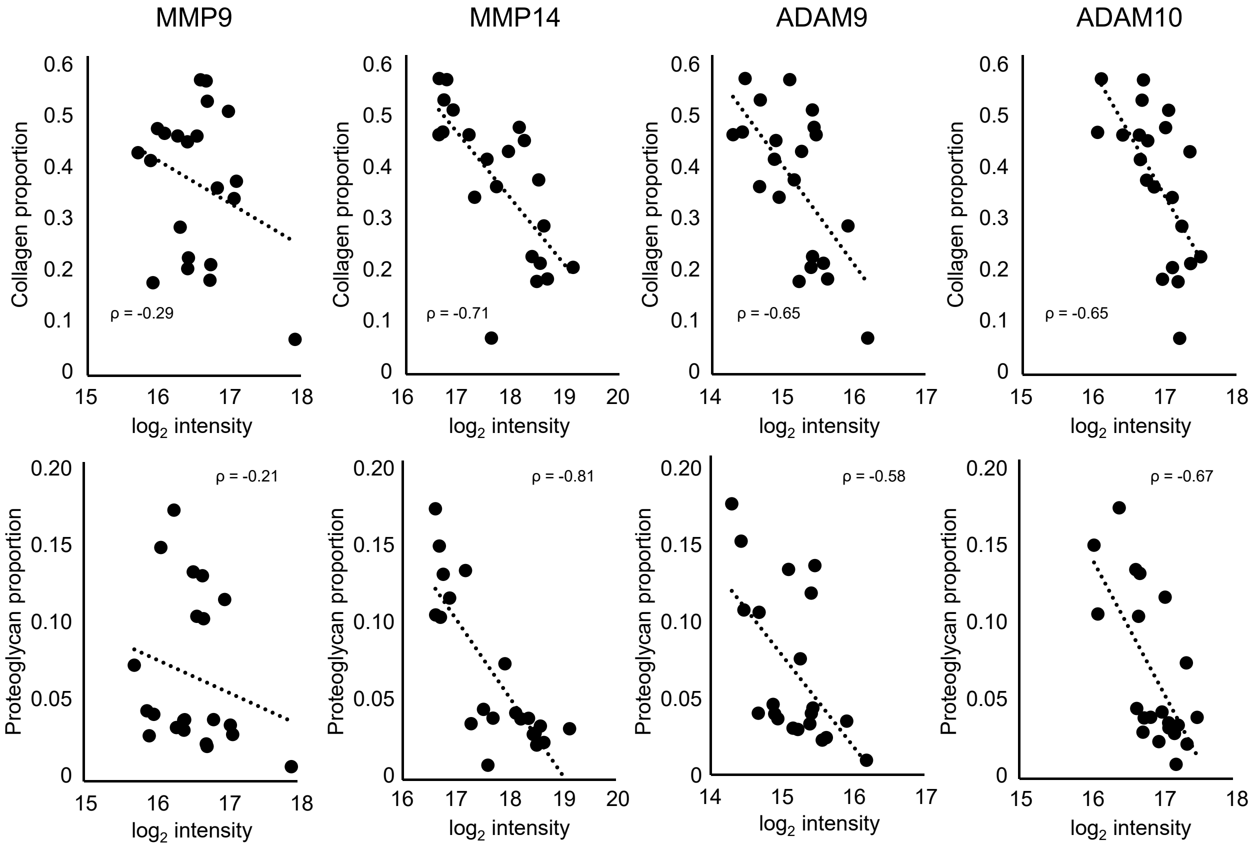

Supple. Fig .3

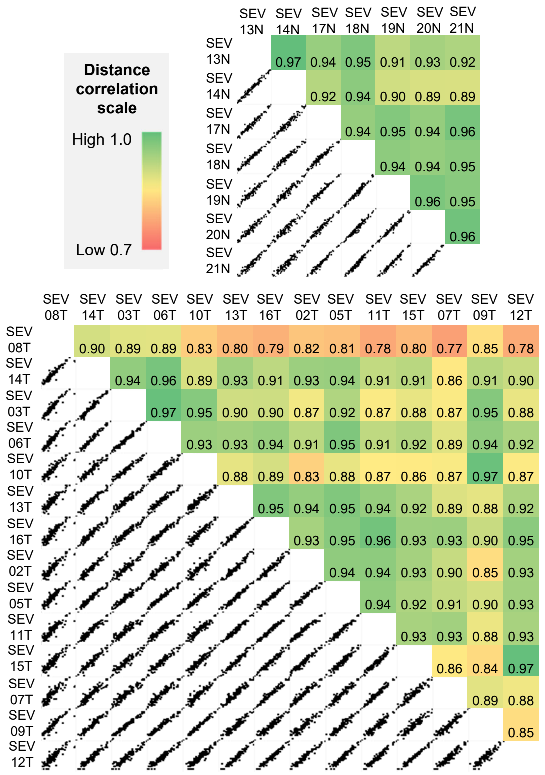

Supple. Fig. 4

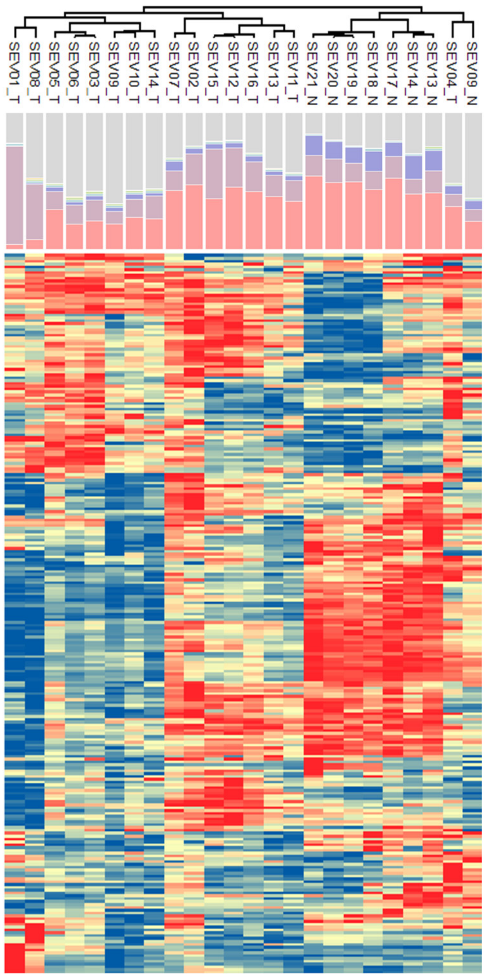

Supple. Fig. 5

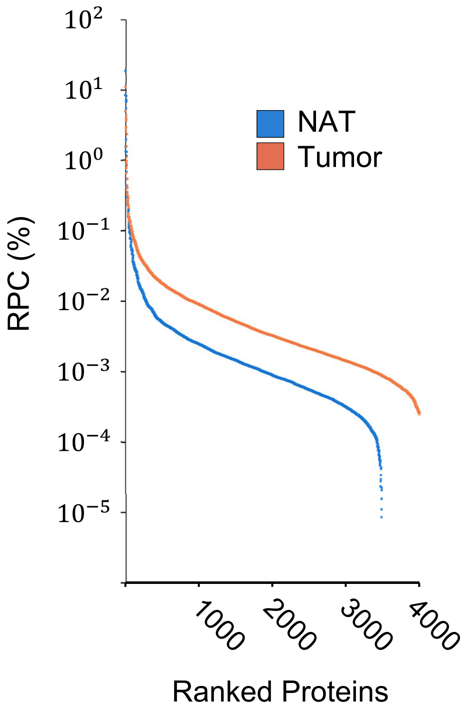

Supple. Fig. 6

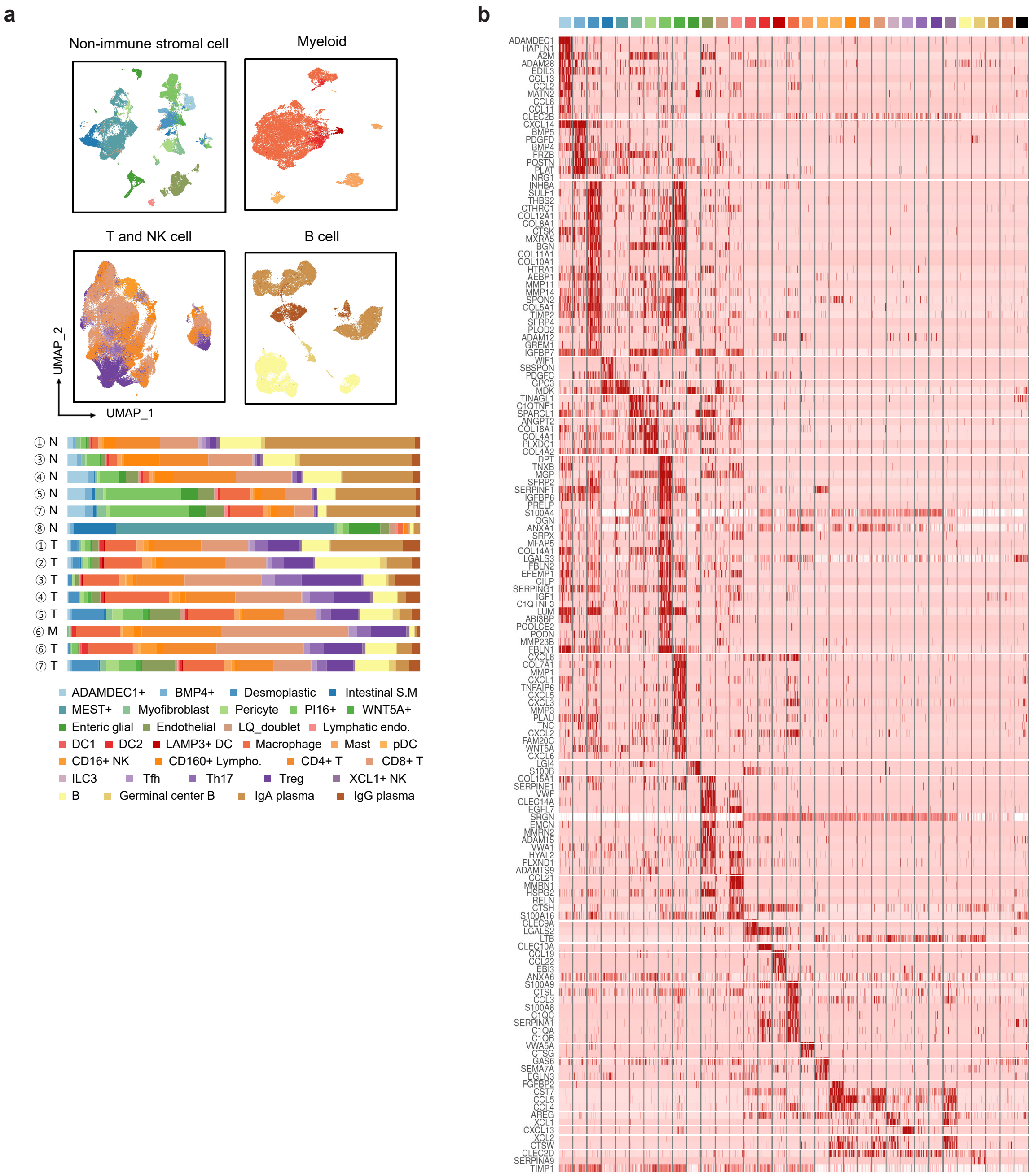
a
b
Non-immune stromal cell
Myeloid
T and NK cell
B cell
UMAP_2
UMAP_1
① N
③ N
④ N
⑤ N
⑦ N
⑧ N
① T
② T
③ T
④ T
⑤ T
⑥ M
⑥ T
⑦ T
ADAMDEC1+
BMP4+
Desmoplastic
Intestinal S.M
MEST+
Myofibroblast
Pericyte
PI16+
WNT5A+
Enteric glial
Endothelial
LQ_doublet
Lymphatic endo.
DC1
DC2
LAMP3+ DC
Macrophage
Mast
pDC
CD16+ NK
CD160+ Lympho.
CD4+ T
CD8+ T
ILC3
Tfh
Th17
Treg
XCL1+ NK
B
Germinal center B
IgA plasma
IgG plasma

Supplementary Figure Legends

Supple. Fig. 1 | Characterization of patient-derived tissue (pd-tissue) a) Cellular location annotation of top 100 enriched protein of reference patient derived ECM (pdECM) sample. b) Quantitative abundance of matrisome proteins in equally mixed internal reference pd-tissue sample from the publicly accessed data(1). The number of detected proteins were expressed in the parentheses. c) Cellular location annotation of top 100 enriched protein of reference pd-tissue sample.

Supple. Fig. 2 | Correlation between collagen/proteoglycan and metalloproteinases The scatter plot of collagen or proteoglycan proportion and metalloproteinases (MMP9, MMP14, ADAM9, ADAM10). $\log_2$ intensity of metalloproteinases and the proportion of collagen or proteoglycan in each sample were shown.

Supple. Fig. 3 | Matrisome profile correlation among pdECM Distance correlation coefficient values and scatter plot of protein intensities among pdECM samples.

Supple. Fig. 4 | Matrisome profiles including excluded samples Hierarchical clustered heatmap with a matrisome profile was shown. Stacked bar plot shows the RPC of each category of matrisome among pdECM samples.

Supple. Fig. 5 | Protein distribution profiles in NAT and tumor pdECM Protein distributions in normal and tumor groups. Detected proteins were ranked according to RPC.

Supple. Fig. 6 | Matrisome markers of heterogenous cell types in integrated scRNA-seq dataset a) Distribution of each cell types in each dataset. b) Matrisome markers of each cell types.

Reference

1. Vasaikar S, Huang C, Wang X, Petyuk VA, Savage SR, Wen B*, et al.* Proteogenomic analysis of human colon cancer reveals new therapeutic opportunities. Cell **2019**;177:1035-49. e19
