# Supplementary material for: Differential cellular origins of the extracellular matrix of tumor and normal tissues according to colorectal cancer subtypes
Source: Br J Cancer. 2025 Mar 3;132(9):770–82. doi: 10.1038/s41416-025-02964-z (PMC12041468; doi:10.1038/s41416-025-02964-z)
Supplement: Supplementary file 2 — Supplementary table legend [file 41416_2025_2964_MOESM2_ESM.pdf]

Supplementary table legend

a) Clinical information for 16 patients. Information about stage, nearestCMS, primary site, and mutation status included. b) TMT set composition. 9 samples were analyzed with each set. c) Normalized intensity data of all detected proteins for all samples. Further analyses were processed after the additional normalization for batch correction. Relative abundance of every proteins in each sample were calculated by dividing the intensity of second reference of each set. Then, for each protein, the average of four intensities from the reference of each of four sets was multiplied for all samples. Finally, log2-transformed values were used. d) Matrisome protein intensity coverage of the proteomic data of our study and native tissue data from Vasaikar et. al. e) The list of differentially expressed matrisome proteins (DEPs). 110 normal enriched DEPs and 28 tumor enriched DEPs are included. f) Cellular origins of DEPs using single-cell sequencing. (Methods in detail)
